# Treatment-Free Remission in Ph+ ALL Without Allogeneic Stem Cell Transplantation: Current Evidence and Future Directions

**DOI:** 10.3390/cancers17152457

**Published:** 2025-07-25

**Authors:** Martina Canichella, Malgorzata Monika Trawinska, Carla Mazzone, Paolo de Fabritiis, Elisabetta Abruzzese

**Affiliations:** 1 Hematology, St. Eugenio Hospital, ASL Roma2, 00144 Rome, Italy; martina.canichella@aslroma2.it (M.C.); trawinskamm@hotmail.com (M.M.T.); c.mazzone@virgilio.it (C.M.); paolo.defabritiis@aslroma2.it (P.d.F.); 2 Department of Biomedicina e Prevenzione, Tor Vergata University, 00133 Rome, Italy

**Keywords:** acute lymphoblastic leukemia Philadelphia positive (Ph+ ALL), tyrosin kinase inhibitors (TKIs), blinatumomab, treatment-free remission (TFR)

## Abstract

Over the past two decades, the treatment landscape of Philadelphia chromosome-positive acute lymphoblastic leukemia (Ph+ ALL) has undergone a profound transformation. Once considered the subtype with the worst prognosis, Ph+ ALL is now associated with the possibility of long-term survival in a significant proportion of patients. This dramatic improvement has been driven by the advent of tyrosine kinase inhibitors (TKIs) and, more recently, by the incorporation of blinatumomab, a bispecific T-cell engager antibody, into frontline therapeutic strategies. In this evolving context, two major areas have become the focus of clinical investigation: on the one hand, the identification of high-risk patients who truly benefit from allogeneic hematopoietic stem cell transplantation (allo-HSCT); on the other, the characterization of patients who can achieve durable responses without transplantation and who may be candidates for treatment discontinuation of TKIs. This review aims to summarize the current evidence supporting the concept of treatment-free remission (TFR) in Ph+ ALL.

## Simple Summary

The treatment landscape of Ph+ ALL has undergone two transformative shifts that have markedly improved patient outcome. First, the advent of TKIs enabled effective and well-tolerated treatment, even in patients ineligible for allogeneic stem cell transplantation. Second, the incorporation of immunotherapy—particularly blinatumomab—in combination with TKIs has led to deep molecular remissions and prolonged survival. As a result, transplantation is now increasingly reserved for patients with high-risk features, such as adverse molecular profiles (e.g., IKZF1^plus^) or persistent minimal residual disease (MRD). Meanwhile, non-transplanted patients are often maintained on indefinite TKI therapy, raising concerns regarding long-term toxicity, psychological burden, and healthcare costs. Emerging retrospective data suggest that treatment-free remission (TFR) may be feasible in a subset of long-term survivors, including those treated in the pre-blinatumomab era. The aim of this perspective paper is to explore the actual feasibility of TFR in Ph+ ALL patients who have not undergone allogeneic transplantation.

## 1. Introduction

Philadelphia chromosome-positive acute lymphoblastic leukemia (Ph+ ALL) is characterized by the presence of the t(9;22)(q34;q11) translocation, which generates the BCR::ABL1 fusion gene. This chimeric gene leads to constitutive activation of the tyrosine kinase signaling pathway, driving leukemogenesis. The incidence of Ph+ ALL increases with age: it is rare in children (2–5%), it accounts for approximately 20–25% of adult cases, and becomes the most frequent genetic subtype in the elderly, where it exceeds 50% [1].

Before the advent of tyrosine kinase inhibitors (TKIs) in 2000, Ph+ ALL was associated with the poorest prognosis among ALL subtypes, with long-term survival rates ranging from 10% to 20%, particularly for patients who were not eligible for allogeneic hematopoietic stem cell transplantation (allo-HSCT) [2,3,4].

Two major therapeutic breakthroughs have dramatically improved outcomes in Ph+ ALL. The first was the introduction of TKIs, with the first in class imatinib, followed by more potent second- and third-generation agents such as dasatinib and ponatinib, which have further enhanced response rates and durable remissions. The second turning point has been the introduction of immunotherapy—particularly the combination of TKIs with blinatumomab, a bispecific CD3-CD19 monoclonal antibody. This approach has enabled chemotherapy-free, immune-based strategies, further improving survival outcomes [5,6].

These advances have profoundly altered the therapeutic landscape of Ph+ ALL, raising new clinical questions. A major issue now is identifying which patients will truly benefit from allo-HSCT, an increasingly nuanced decision. Moreover, in patients who will not undergo transplantation, the feasibility of discontinuing TKI therapy has become a relevant clinical consideration. Current clinical practice still tends to favor indefinite TKI administration, but long-term TKI exposure, especially to higher generation drugs, raises serious safety concerns.

For this reason, inspired by the paradigm established in chronic myeloid leukemia (CML), where treatment-free remission (TFR) is an accepted and achievable goal, there is growing interest in exploring TFR in Ph+ ALL as well [7]. This review aims to examine the current evidence supporting TFR in Ph+ ALL focusing on the subset of patients for whom this strategy may be appropriate and to highlight the optimal timing and monitoring strategies during TFR.

## 2. Advanced Treatment Strategies in Ph+ ALL

As mentioned above, the treatment landscape of Ph+ ALL has been profoundly transformed by the introduction of TKIs, beginning with imatinib in 2000, followed by dasatinib in 2006, and ponatinib in 2010. Initially, imatinib was combined with intensive chemotherapy regimens that, despite yielding high complete hematological remission (CHR) rates, were associated with significant toxicity and a non-negligible risk of early death [8,9,10,11]. Over time, the Italian GIMEMA (Gruppo Italiano Malattie Ematologiche dell’Adulto) group developed a chemo-free induction approach based on TKI plus glucocorticoids and central nervous system prophylaxis [12,13,14]. This strategy led to CR rates of roughly 100%. At the same time, the TKI-based approach (typically with imatinib or dasatinib) combined with chemotherapy, adopted by multiple groups including MD Anderson Cancer Center (MDACC), has demonstrated 5-year overall survival (OS) rates of approximately 50% [15,16]. Subsequently, the introduction of ponatinib, a third-generation pan-TKI able to overcome the T315I mutation in the ABL1 kinase domain, has further improved outcomes [17]. In a prospective study by MDACC evaluating ponatinib in combination with blinatumomab in 86 newly diagnosed Ph+ ALL patients, with a median follow-up of 80 months, the 6-year OS reached 75% [18,19,20]. Indeed, analyses and meta-analyses of clinical trials have confirmed the superiority of ponatinib over earlier TKIs and highlighted that those patients achieving CMR within the first 3 months had the most favorable survival outcomes [21]. Among these, ponatinib exposure emerged as an independent prognostic factor for improved OS [22]. In parallel, evidence supporting the efficacy of blinatumomab in relapsed/refractory (R/R) Ph+ ALL began to emerge. Rambaldi et al. conducted a propensity score-matched analysis comparing 45 R/R patients treated with blinatumomab to 55 patients receiving standard of care (SOC) [23]. The blinatumomab cohort achieved higher rates of CHR (36% vs. 25%) and OS. Similarly, the final analysis of the ALCANTARA trial by Martinelli et al. provided robust evidence of blinatumomab’s capacity to induce deep responses in R/R Ph+ ALL [24]. In this study, 16 of 45 patients (36%) achieved CHR, and 14 of them also reached MRD negativity. These encouraging results catalyzed the incorporation of blinatumomab into frontline strategies. The GIMEMA LAL2116 trial was the first to prospectively explore a chemo-free immunotherapy-based approach combining dasatinib and blinatumomab [25]. CHR was achieved in 98% of patients. CMR at the end of dasatinib-only induction (day 85) was 29%, but increased to 60% after two cycles of blinatumomab. Long-term follow-up at 53 months confirmed durable outcomes, with DFS, OS, and EFS rates of 75.8%, 80.7%, and 74.6%, respectively [26]. The combination of blinatumomab and ponatinib was further investigated in a phase II trial by Shorts et al., enrolling 44 patients. CMR rates after one cycle and overall were 64% and 85%, respectively [27]. Among 25 evaluable patients, 22 (88%) had undetectable MRD by next-generation sequencing (NGS). This subgroup showed exceptional outcomes, with estimated 3-year progression-free survival (PFS) and OS rates of 95%. Building on these findings, the GIMEMA group designed the phase III LAL2820 trial, comparing the blinatumomab-ponatinib combination to an imatinib-plus-chemotherapy control arm. Preliminary results from the experimental arm, presented at the 66th ASH Annual Meeting, revealed a CHR rate of 98% and an estimated 18-month OS of 91.6%, with a median follow-up of 6.4 months (range 0.1–32.3) [28]. Taken together, these data suggest a paradigm shift in the therapeutic algorithm of Ph+ ALL. The future challenge lies in identifying patients who may truly benefit from a chemo-free, TKI- and immunotherapy-based strategy and those who still require allo-HSCT consolidation.

Emerging tools from molecular biology, developed alongside clinical trials, could drive this patient stratification. Notably, the presence of additional genetic alterations (such as the IKZF1^plus^ profile) and persistent MRD at defined time-points are increasingly recognized as relevant prognostic markers in tailoring patient treatment intensification [29].

## 3. Treatment-Free Remission in Ph+ ALL

Given the promising long-term survival rates observed with novel treatment strategies combining TKIs and blinatumomab, there is growing interest in the possibility of discontinuing TKI therapy in Ph+ ALL patients who do not undergo allo-HSCT. This issue is further supported by real-world clinical observations that a subset of patients, previously treated with pre-TKI and blinatumomab era, maintained CMR while continuing on long-term TKI therapy.

Based on these considerations, several study groups have begun to retrospectively investigate the outcomes of Ph+ ALL patients who, primarily due to the emergence of treatment-related adverse events, have discontinued TKI therapy (Table 1).

In a retrospective analysis about patients treated at the MD Anderson Cancer Center (MDACC), Samra and colleagues described a cohort of 9 Ph+ ALL patients who discontinued TKI therapy following a median duration of 70 months on treatment and 47 months of sustained deep molecular remission (DMR) [30]. Molecular relapse occurred in 3 out of 9 patients (33%), with a median time of 6 months. Two of these relapsed cases were re-treated with TKI and successfully regained DMR. With a median follow-up of 49 months from TKI discontinuation, the median TFR duration was not reached, and the estimated TFR rate at 4 years was 65%. Interestingly, the duration of DMR before discontinuation tended to be longer among those who remained relapse-free compared to those who relapsed (median 58 vs. 22 months; *p* = 0.096), suggesting that prolonged molecular suppression may be associated with a more durable TFR.

Another relevant experience comes from the Italian Campus ALL group, which reported long-term outcomes of 18 Ph+ ALL patients who had discontinued TKI therapy [31]. In this retrospective analysis by Dragani et al., the median duration of TFR following TKI cessation was 14 months. Prior therapy consisted of imatinib in nine patients (50%), dasatinib in four (22%), sequential nilotinib/imatinib as per the GIMEMA LAL1408-6 protocol in one patient (6%), and ponatinib in four (22%). Molecular relapse occurred in 5 out of 18 patients (28%), with a median time to relapse of 4 months. Among them, 4/5 patients resumed the same TKI and successfully regained CMR. With a median follow-up of 10 years (range 0.8–26) neither median OS nor relapse-free survival (RFS) had been reached. The estimated 5-year OS and RFS were 79% and 63%, respectively, further supporting the feasibility of TFR in a subset of patients with sustained molecular response.

More recently, Kugler et al. conducted a retrospective chart review using the MDACC database and analyzed 14 patients with Ph+ ALL who discontinued TKI therapy after a median of 60 months of treatment [32]. A total of 12/14 patients (86%) received hyperfractionated cyclophosphamide, vincristine, doxorubicin, and dexamethasone alternating with high-dose methotrexate and cytarabine combined with either imatinib (14%), dasatinib (43%), or ponatinib (29%); two patients received blinatumomab and ponatinib. Prior to discontinuation, the median duration of sustained CMR was 46.1 months (range: 2.7–121.3 months). With a median follow-up of 42.5 months post-TKI discontinuation, three patients (21%) experienced relapse—two at the molecular level and one with morphologic disease. The median time to relapse was 6.4 months (range: 4–16 months). Among the relapsed cases, two patients reinitiated TKI therapy and subsequently achieved CMR again. Remarkably, none of the patients who had maintained CMR for more than 48 months prior to TKI cessation experienced relapse, suggesting a possible protective threshold. The median TFR duration was not reached, with estimated TFR rates of 85% at 12 months and 75% at 36 months, underscoring the potential durability of remission in carefully selected patients.

## 4. Discussion

The long-term management of Ph+ ALL with TKIs remains a topic of considerable debate, both in the non-transplant and post-transplant settings. In the latter scenario, emerging evidence supports the role of continued TKI therapy as a maintenance strategy aimed at reducing the risk of relapse [33]. Although no formal international guidelines currently exist regarding TKI use post-transplantation in Ph+ ALL, several expert consensus statements and position papers provide practical recommendations. The principal recommendation is to initiate post-transplant maintenance therapy with TKIs in all patients with Ph+ ALL regardless of MRD status, typically beginning around day +100 post allo-HSCT, assuming adequate hematologic recovery and absence of severe graft-versus-host disease [34].

Among available TKIs, ponatinib appears to be associated with the most favorable survival outcomes and safety profile [35,36]. However, these conclusions are primarily drawn from retrospective real-world studies, and randomized prospective data are currently lacking. With regard to treatment duration, expert recommendations suggest that post-transplant maintenance should not be shorter than 24 months. In the setting of patients who did not undergo allo-HSCT, the possibility to stop TKI is an attractive option considering the possible onset of side effects. When analyzing the three clinical experiences discussed above, some elements clearly support the feasibility of TFR in Ph+ ALL, while others highlight significant limitations. The primary concerns lie in the retrospective design of these studies, the limited number of patients included, and the heterogeneity of frontline therapies—ranging from TKI plus chemotherapy regimens to combinations of TKIs with blinatumomab.

Despite these limitations, the available data suggest that TFR may be a realistic option for a highly selected subgroup of patients. Those who achieve and maintain deep molecular remission (DMR) for a prolonged period appear to be the best candidates. Across all reports, early relapse—typically occurring within the first year following TKI discontinuation—is a consistent observation, underscoring the need for intensive molecular monitoring during this critical time frame.

Patient selection for TFR should not be based solely on molecular response kinetics. In fact, baseline risk stratification—potentially including the presence of additional high-risk genetic lesions (e.g., IKZF1^plus^)—might also help refine prognostic assessment. Ultimately, the integration of molecular response depth, its duration, and underlying genomic profile will be essential to identify candidates who can safely attempt TKI discontinuation without compromising long-term outcomes. Prospective clinical trials are critically needed to validate these preliminary findings and to establish standardized criteria for TKI discontinuation in Ph+ ALL.

## 5. Conclusions and Future Directions

In the near future, TFR may emerge as a viable therapeutic goal for selected patients with Ph+ ALL who are not candidates for allo-HSCT. The refinement of molecular techniques for MRD monitoring will be instrumental in driving TKI discontinuation in order to achieve an early reinitiation in the event of molecular relapse. At the same time, an increasing number of patients will be treated front-line with TKI plus blinatumomab, a monoclonal antibody with known immunomodulatory properties. This immunologic effect may contribute to maintaining disease control following TKI discontinuation, thereby enhancing the safety of TFR in selected cases.

Altogether, these considerations highlight that, despite the remarkable advances achieved in the treatment of Ph+ ALL, there remains significant potential for further optimization. As therapeutic strategies continue to evolve, a less conservative approach may be the key to balancing efficacy with long-term safety. The ultimate goal is not only to prolong survival, but also to preserve, and ideally enhance patients’ quality of life. Moving toward sustained remissions with reduced treatment burden represents a meaningful step forward, and TFR aligns directly with this goal.

## Figures and Tables

**Table 1:** Three main retrospective studies regarding TFR in Ph+ ALL.

Study	N. of Patients	Median TKI Duration (mo)	Median CMR Duration (mo)	Relapse Rate (%)	Median Time to Relapse (mo)	Re-Achieved CMR After Relapse	TFR at 12 Months (%)	TFR at 36 Months (%)	5-Year OS (%)	5-Year RFS (%)
Samra et al.	9	70	47	33	6	2/3	Not reported	Not reported	Not reported	Not reported
Dragani et al.	18	Not specified	Not specified	28	4	4/5	Not reported	Not reported	79	63
Kugler et al.	14	60	46.1	21	6.4	2/3	85	75	Not reported	Not reported

N: number; TKI: Tyrosin kinase inhibitors; CMR: complete molecular remission; TFR: treatment-free remission; OS: Overall survival; RFS: relapse-free survival.
